# Supplementary material for: Anti-nucleocapsid SARS-CoV-2 antibody seroprevalence in previously infected persons with immunocompromising conditions—United States, 2020–2022
Source: PLoS One. 2025 Jan 8;20(1):e0313620. doi: 10.1371/journal.pone.0313620 (PMC11709286; doi:10.1371/journal.pone.0313620)
Supplement: S1 Table — (DOCX) [file pone.0313620.s001.docx]

**S1 Table. ICD10 codes used to categorize IC types**

| **IC Categories** | **ICD-10 Codes** | **Conditions** |
| --- | --- | --- |
| Solid malignancy | C00–C80, C7A, C7B, D3A, Z51.0, and Z51.1 | Malignant neoplasms; Malignant neuroendocrine tumors; Secondary neuroendocrine tumors; Benign endocrine tumors; Encounter for antineoplastic radiation therapy; Encounter for antineoplastic chemotherapy and immunotherapy |
| Hematologic malignancy | C81–C86, C88, C90–C96, D46, D61.0, D70.0, D61.2, D61.9, and D71 | Malignant neoplasms of lymphoid, hematopoietic, and related tissue; Myelodysplastic syndromes; Other aplastic anemias and other bone marrow failure syndromes; Constitutional aplastic anemia; Functional disorders of polymorphonuclear neutrophils |
| Rheumatologic or inflammatory disorder | D86, E85 (except E85.0), G35, J67.9, L40.54, L40.59, L93.0, L93.2, L94, M05–M08, M30, M31.3, M31.5, M32–M34, M35.3, M35.8, M35.9, M46, and T78.40 | Sarcoidosis; Amyloidosis; Multiple sclerosis; Hypersensitivity pneumonitis due to unspecified organic dust; Psoriatic juvenile arthropathy; Other psoriatic arthropathy; Lupus erythematosus; Other localized connective tissue disorders; Rheumatoid arthritis; Enteropathic arthropathies; Juvenile arthritis; Polyarteritis nodosa and related conditions; Wegener's granulomatosis; Giant cell arteritis with polymyalgia rheumatica; Systemic lupus erythematosus (SLE); Dermatopolymyositis; Systemic sclerosis [scleroderma]; Polymyalgia rheumatica; Other specified systemic involvement of connective tissue; Systemic involvement of connective tissue, unspecified; Other inflammatory spondylopathies; Allergy, unspecified |
| Other intrinsic immune condition or immunodeficiency | D27.9, D61.09, D72.89, D80, D81 (except D81.3), D82–D84, D89 (except D89.2), K70.3, K70.4, K72, K74.3–K74.6 (except K74.60 and K74.69), N04, and R18 | Benign neoplasm of ovary; Fanconi's anemia; Other specified disorders of white blood cells; Immunodeficiency with predominantly antibody defects; Combined immunodeficiencies (except Adenosine deaminase [ADA] deficiency); Immunodeficiency associated with other major defects; Common variable immunodeficiency with predominant abnormalities of B-cell numbers and function; Lymphocyte function antigen-1 [LFA-1] defect; Diseases of the blood and blood-forming organs and certain disorders involving the immune mechanism (except Hypergammaglobulinemia; unspecified); Alcoholic cirrhosis of liver; Alcoholic hepatic failure; Hepatic failure not elsewhere classified; Primary biliary cirrhosis; Secondary biliary cirrhosis; Biliary cirrhosis unspecified; Other and unspecified cirrhosis of liver; Nephrotic syndrome; Ascites |
| Organ or stem cell transplant | T86 (except T86.82–T86.84, T86.89, and T86.9), D47.Z1, Z48.2, Z94, and Z98.85 | Complications of transplanted organs and tissue; Post-transplant lymphoproliferative disorder (PTLD); Encounter for aftercare following organ transplant; Transplanted organ and tissue status; Transplanted organ removal status |
