# Supplementary material for: Anti-nucleocapsid SARS-CoV-2 antibody seroprevalence in previously infected persons with immunocompromising conditions—United States, 2020–2022
Source: PLoS One. 2025 Jan 8;20(1):e0313620. doi: 10.1371/journal.pone.0313620 (PMC11709286; doi:10.1371/journal.pone.0313620)
Supplement: S2 Table — (DOCX) [file pone.0313620.s002.docx]

**S2 Table. Adjusted odds ratios for anti-N seroprevalence in various time periods following a positive SARS-CoV-2 viral test result by immunocompromising conditions (IC)— United States, July 2020–February 2022**

|  | Adjusted Odds Ratios* | | | | | | | | | |
| --- | --- | --- | --- | --- | --- | --- | --- | --- | --- | --- |
|  | 14–90 days | | | 21–90 days | | | 28–90 days | | |  |
| IC status, prior 5 years | OR | LL | UL | OR | LL | UL | OR | LL | UL |  |
| *No IC* | *reference* | | | *reference* | | | *reference* | | |  |
| Any IC | 0.61 | 0.40 | 0.93 | 0.65 | 0.42 | 1.02 | 0.82 | 0.53 | 1.27 |  |
| *Solid malignancy*  ICD10 codes: C00–C80, C7A, C7B, D3A, Z51.0, Z51.1 | 0.03 | 0.00 | 0.51 | 0.08 | 0.02 | 0.40 | 0.11 | 0.03 | 0.41 |  |
| *Hematologic malignancy*  ICD10 codes: C81–C86, C88, C90–C96, D46, D61.0, D70.0, D61.2, D61.9, D71 | 0.42 | 0.09 | 1.98 | 0.53 | 0.10 | 2.79 | 0.47 | 0.09 | 2.53 |  |
| *Rheumatologic or inflammatory disorder*  ICD10 codes: D86, E85 [except E85.0], G35, J67.9, L40.54, L40.59, L93.0, L93.2, L94, M05–M08, M30, M31.3, M31.5, M32–M34, M35.3, M35.8, M35.9, M46, T78.40 | 0.67 | 0.40 | 1.11 | 0.69 | 0.40 | 1.18 | 0.64 | 0.36 | 1.14 |  |
| *Organ or stem cell transplant*  ICD10 codes: T86 [except T86.82–T86.84, T86.89, and T86.9], D47.Z1, Z48.2, Z94, Z98.85 | - | | | - | | | - | | |  |
| *Other intrinsic immune condition*  ICD10 codes: D27.9, D61.09, D72.89, D80, D81 [except D81.3], D82–D84, D89 [except D89.2], K70.3, K70.4, K72, K74.3–K74.6 [except K74.60 and K74.69], N04, R18 | 0.52 | 0.27 | 0.97 | 0.59 | 0.30 | 1.16 | 0.52 | 0.26 | 1.06 |  |

*OR = Odds Ratio, LL = Lower limit of 99% Confidence interval, UL = Upper limit of 99% Confidence interval, - = results excluded due to imprecise estimates, shaded cells indicate statistical significance at 99% confidence*

**adjusted for age, sex, metro status, and SVI tertile*
