# Supplementary material for: Anti-nucleocapsid SARS-CoV-2 antibody seroprevalence in previously infected persons with immunocompromising conditions—United States, 2020–2022
Source: PLoS One. 2025 Jan 8;20(1):e0313620. doi: 10.1371/journal.pone.0313620 (PMC11709286; doi:10.1371/journal.pone.0313620)
Supplement: Attachment — Submitted filename: Plos one Response to Reviewers.docx [file pone.0313620.s003.docx]

Journal requirements:

When submitting your revision, we need you to address these additional requirements.

1. Please ensure that your manuscript meets PLOS ONE's style requirements, including those for file naming. The PLOS ONE style templates can be found at

<https://journals.plos.org/plosone/s/file?id=wjVg/PLOSOne_formatting_sample_main_body.pdf> and

<https://journals.plos.org/plosone/s/file?id=ba62/PLOSOne_formatting_sample_title_authors_affiliations.pdf>

We heve ensured our paper follows PLOS ONE's style requirements.

2. We note that the grant information you provided in the ‘Funding Information’ and ‘Financial Disclosure’ sections do not match.

When you resubmit, please ensure that you provide the correct grant numbers for the awards you received for your study in the ‘Funding Information’ section.

Thank you for bringing this issue to our attention. We did not receive specific grants for this work; it was part of our routine work at CDC. I have updated the funding information to better reflect this.

3. For studies involving third-party data, we encourage authors to share any data specific to their analyses that they can legally distribute. PLOS recognizes, however, that authors may be using third-party data they do not have the rights to share. When third-party data cannot be publicly shared, authors must provide all information necessary for interested researchers to apply to gain access to the data. (<https://journals.plos.org/plosone/s/data-availability#loc-acceptable-data-access-restrictions>)

For any third-party data that the authors cannot legally distribute, they should include the following information in their Data Availability Statement upon submission:

1) A description of the data set and the third-party source

2) If applicable, verification of permission to use the data set

3) Confirmation of whether the authors received any special privileges in accessing the data that other researchers would not have

4) All necessary contact information others would need to apply to gain access to the data

Thank you for bringing this issue to our attention. We have included futher information in the submission.

4. Please include captions for your Supporting Information files at the end of your manuscript, and update any in-text citations to match accordingly. Please see our Supporting Information guidelines for more information: <http://journals.plos.org/plosone/s/supporting-information>.

n/a

5. Please review your reference list to ensure that it is complete and correct. If you have cited papers that have been retracted, please include the rationale for doing so in the manuscript text, or remove these references and replace them with relevant current references. Any changes to the reference list should be mentioned in the rebuttal letter that accompanies your revised manuscript. If you need to cite a retracted article, indicate the article’s retracted status in the References list and also include a citation and full reference for the retraction notice.
We have checked and did not see any issues with our references being retracted. If you are referring to a specific reference, please let us know.


**Comments to the Author**

Reviewer #1: Dear Editor,

The authors have done a nice job in putting up the manuscript and presenting it in a concise and clear manner. The topic of immune responses to a respiratory infection in the immunocompromised is apt and the findings may offer insight for public health approach to such infections.
Having said that the authors can respond and clarify a few observations in the manuscript.

Thank you for your thoughtful review! We hope the added clarifications (detailed below) thoroughly address your questions.

1. The nucleocapsid antibodies were assayed using two kits from different manufactures (Abbott and Roche). Commercial assays used to detect anti-N antibodies have highly variable performances with significant loss of sensitivity with time postinfection (the performance specifications of the kits are not provided). However, the authors have not shared information on the distribution of the two assays between the immunocompromised and the non-immunocompromised- and whether this influenced the study findings.

Thank you for pointing this important point. In re-visiting our data for this analysis, Abbott was the only anti-N assay used. (Roche was used at another commercial lab network contributing to NCLS for specimens not included in this analysis.)

We have removed reference to Roche and added performance specifications for Abbott. (Lines 85-88).

2. The vaccination status of participants is unknown. A study reported that anti-N antibody responses were lower in plasma after SARS-CoV-2 infection in vaccinated patients compared with patients infected before vaccination or infected without vaccination. Again, since information is not provided one cannot tell what impact this would have had on the study findings especially if those who were IC had been encouraged to get the shot and ended with higher proportion of vaccinated compared with non-IC.

Thank you for pointing out this limitation. We have added discussion of this to lines 194-195.

3. Severity of SARS-CoV-2 infections is not provided, and this would have had an impact on the response and duration of anti-N antibodies. Did the IC have more severe infections than the non-IC or the vice versa?

Thank you for this great question. We are also curious to know, but unfortunately, severity of SARS-CoV-2 infection was not included in our data. We have changed our third limitation to discuss our lack of severity information on both IC and COVID-19. (lines 190-194)

Reviewer #2: In the manuscript “Anti-Nucleocapsid SARS-CoV-2 Antibody Seroprevalence in Previously Infected Persons with Immunocompromising Conditions — United States, 2020–2022” by Bratcher et al, the authors analyze data from Quest looking at the ability of immunocompromised and non-immunocompromised patient’s ability to mount an infection-induced antibody response to SARS-CoV-2 infection. Overall, I think this paper was well researched but I believe there are a few areas that need improvement before publication. See specific questions and notes below:

Thank you for your thoughtful review! We hope the edits and added information detailed below alleviate any concerns.

Methods:
-Authors should define ICD10 codes used to categorize patients. This could be a supplemental figure, but each code used should be defined. “Other intrinsic immune conditions” should also be defined.

Thank you for this suggestion. We have added a supplemental table detailing this.

-Methodology for serology was defined but specific viral test was not. Please include.

Thank you for pointing out this omission. We have added more information on the anti-N assay used (lines 85-88).

-Statistics – it was mentioned that statistics were not corrected for multiple comparisons, but I they should have been. I would consult with a statistician to be sure.

Using 95% confidence intervals would lead us to expect one type I error (false positive) in our 20 statistical tests (if there were actually no differences i.e. under the null). We have changed to 99% confidence intervals, which would lead us to most likely not having any false positives. Indeed, we lose one significant finding going from 95 to 99% confidence.

Table 2 is updated, along with added/updated discussion in lines 32-34, 115, 147-148, 172-173

Also, you mention p values, but none are presented. Please include them.

Thank you for bringing this to our attention. We have changed the reference to p-values to reference CIs, as this wording better fits what results we display. We opted to keep CIs instead of changing to p-values to retain the ability for readers to see precision of the estimates. (line 115)

-Were previously infected patients (those with positive serology before viral testing) included in analysis or were they left out?

97 patients were positive for anti-N antibodies prior to their first positive PCR. We did not exclude these individuals from the analysis.

We have added a brief note in lines 73-74 to clarify.

-Could you better define sensitivity analysis and what you compared?

Thank you for pointing this out. We hope the reworded lines 106-107 make our methods clearer.

Results:
-It would be helpful if you include a demographics table that included the typical age, sex, SVI, metropolitan data, how many patients were previously infected before viral test, etc for both non and IC groups.

Good suggestion! We have added this information in Table 1. Text is updated in lines 133-143 to discuss the distributions across the No IC and Any IC groups.

-Table 1: If using “LL” and “UL” you must define it under the table. You could also consider turning these numbers into a graphic by graphing the range and noting the OR with a symbol. Graphing in this way and including a line at 1 could help distinguish the significant findings. I assume the blue highlight is to also distinguish the significant values, but that should be defined somewhere if you decide to stick to this table format.

Thank you for pointing this out. We have added more detail below the table.

We have used the suggested figures when presenting our finding internally. They were helpful, but required 4 separate views, one for each time point. Unless there are strong objections, we will opt to keep the table as is to keep results in one place.

-Sensitivity analysis is mentioned below the table as being similar but you need to define that data.

Thank you for noting this omission. We have added a supplemental Table to display these results.

Discussion:
-You mention you looked at sensitivity analysis of other time periods (14, 21, 28-90)[lines 129-130] but this wasn’t in the methods or results. Please provide if you are going to include it in the discussion.

Thank you for noting this omission. We have added a supplemental Table to display these results.

Please note that including this data with 99% intervals (as opposed to 95% CIs) changes our findings. We have updated the referent text in lines 161-163 and lines 178-180.

-You mention increased reinfection rates [line 132] in the discussion but don’t provide that data. You could include this in the demographics table I mention above.

We were able to see some reinfections (n=812), but unfortunately there were extremely few cases where we had an anti-N result following the reinfection. As mentioned above, we did not feel confident that those without a recorded prior infection actually were not previously infected. Therefore, we’ve added language to the methods (lines 73-74) to clarify.

-Limitations: It would be good to mention that the time frame of looking at ICD10 codes 5 years prior may not accurately represent the patient’s immune status at time of testing.

Yes, this is a good point! We have added it to lines 186-187.
